# Evaluation of the impact of a 8 week exposure to a portable light therapy device, Luminette®, on retinal function assessed by ElectroRetinoGraphy

**DOI:** 10.1016/j.crtox.2025.100253

**Published:** 2025-08-14

**Authors:** Marie de Deus, Charlotte Petit, Eve Cosker, Amandine Luc, Cédric Baumann, Thomas Schwitzer

**Affiliations:** a Pôle Hospitalo-Universitaire de Psychiatrie d’Adulte et d’Addictologie du Grand Nancy, Centre Psychothérapique de Nancy, F-54521 Laxou, France; b Unité de méthodologie, data management et statistique, DRCI, CHRU de Nancy, Nancy, Lorraine, France

**Keywords:** Light therapy, Electroretinography, Major Depressive Disorder, Retinal function, Tolerability

## Abstract

**Background:**

Major depressive disorder (MDD) treatment can be long and difficult to obtain. Thus, alternative non-pharmacological treatments, such as light therapy (LT), are increasingly recommended to treat MDD. For better treatment adherence, portable LT devices have been developed. However, more research is needed to better understand their safety of their use on patients’ physiology. One way to explore it is evaluating the retina function. That is why the aim of the study presented was to assess, thanks to Electroretinography (ERG), the impact on retinal function of an 8 weeks exposure to an active or a placebo LT portable device in MDD patients.

**Method:**

MDD patients were treated with an active portable LT device or with a placebo LT device. The LT device tested was Luminette®. ERG measurements were carried out before the start of the LT treatment and 4 and 8 weeks afterwards.

**Results:**

No significant differences were found in the ERG waveforms between the patients treated with active Luminette® and patients treated with the placebo device.

**Conclusions:**

The use of the Luminette® device for 8 weeks, combined with usual care, did not result in any morphological or quantitative alterations in ERG waveforms in our cohort of MDD patients. This portable LT device would therefore be well tolerated at retinal level in MDD patients.

## Highlights

• Portable LT devices have emerged, but more data on their retinal impact are needed.
• ERG can be used to assess retinal function in MDD patients treated with portable LT.
• ERG showed no retinal alterations, suggesting good tolerance of portable LT in MDD.

## Graphical abstract

**Figure:**
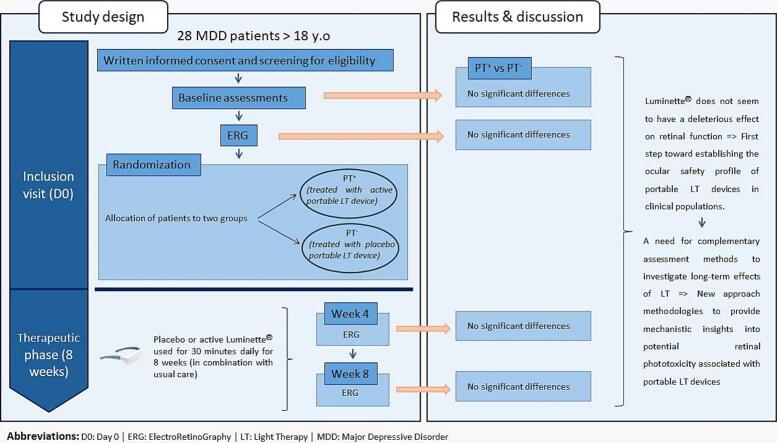

## Background

Major Depressive Disorder (MDD) is a chronic and frequent disorder affecting about 3,8 % of the world's population (World Health Organization, 2023). It is characterized by at least one Major Depressive Episode (MDE) without mania nor hypomania. MDE are periods of at least 2 weeks, where patients suffer from persistent and pervasive depressed mood and/or anhedonia with other psychoaffective symptoms (American Psychiatric Association, 2013).

MDD is considered as one of the most disabling chronic diseases (GBD 2019 Mental Disorders Collaborators, 2022), notably because of associated cognitive dysfunctions (Varghese et al, 2022) and chronic physical disorders (Scott et al., 2016, Wang et al., 2020, Zhang et al., 2023). This causes indirect and direct excess costs (König et al, 2019). Therefore, this disorder is a current major public health problem. However, the response to MDD treatments can be long and difficult to obtain, requiring numerous trials (Malhi and Mann, 2018). In spite of this, the response remains inadequate for around 20 % of MDD patients treated for 2 or more years from episode onset (Day et al, 2021). As a result, depressive symptoms and cognitive dysfunction persist (Mandal et al, 2022), leading to a genuine distress for mistreated MDD patients. These patients suffer from an impaired quality of life and a daily functional disability (Day et al, 2021). Thus, alternative non-pharmacological treatments are increasingly recommended to treat MDD.

Light therapy (LT) is one of them. This treatment has been used for decades in seasonal affective disorder (Westrin and Lam, 2007). More recently, LT has been proven effective and well-tolerated alone or in combination with fluoxetine in nonseasonal MDD patients (Lam et al, 2016). Additionally, LT is safe and inexpensive (Campbell et al. 2017). LT is therefore increasingly recommended, alone or in combination with antidepressants, to treat symptoms of non-seasonal MDD and improve patients’ quality of life (Morton et al., 2021, Menegaz de Almeida et al., 2025).

LT consists of exposing the eyes to a source of artificial light − typically bright light enriched with blue light. By simulating natural light exposure, LT may alleviate depressive symptoms through its action on specific circuits linking the retina to the brain. It acts initially on the retina, in particular on its photosensitive cells − rods, cones and intrinsically photosensitive retinal ganglion cells (ipRGCs) (Li and Li, 2018, Huang et al., 2024). This direct retinal action raises an important concern: whether LT may cause damage to these sensitive retinal structures.

Indeed, light exposure can be harmful to the eyes in the long term, especially when it falls within the high-intensity visible spectrum (390–600 nm). This is the case for blue light, whose peak wavelength falls between 400 and 500 nm (Tosini et al., 2016, Antemie et al., 2023). Blue light – in itself or emitted by everyday objects such as visual display terminals (mobile phones, computers…) or LED lighting – has been linked to visual disturbances, including photophobia (Zivcevska et al., 2018) and eye fatigue (Krigel et al., 2016). It is also considered a risk factor for the development of age-related macular degeneration (AMD) and other retinal disorders (Tosini et al., 2016, Nagai et al., 2015). Exposure to blue light induces both functional damage (Li et al., 2021) and structural damage to retina (Li et al., 2021, Nagai et al., 2015). The underlying mechanism involves photochemical injury, including oxidative stress and mitochondrial apoptosis, primarily affecting the retinal and ocular surface (Ouyang et al., 2020, Tosini et al., 2016). Even though conventional LT lamps rely on blue-light, they do not seem to cause visual damage (Brouwer et al. 2017).

As LT is increasingly used in routine practice for MDD patients, portable LT devices have been developed to improve adherence. However, they may differ from conventional LT lamps in terms of the light they emit − in particular with respect to intensity and duration of exposure – which are factors known to influence the potential phototoxicity of light (Tosini et al., 2016). Additionally, the light emission is closer to the eyes with portable LT devices. Thus, the action of these portable LT devices on the retina may pose a potential risk of harm, unlike conventional LT lamps. It is therefore necessary to evaluate the potential phototoxicity of portable LT devices on the retina to determine whether they are safe for use.

Electroretinography (ERG) is used to assess retinal function. It employs electrodes to record electrophysiological responses from retinal neuronal cells, including rods and cones. In response to low-level visual stimulation, ERG generates characteristic waves whose amplitude and implicit time are analyzed to evaluate the retinal function. There are three main types of ERG, each targeting different retinal regions: Full-field ERG (ffERG) (Robson et al, 2022), Pattern ERG (PERG) (Thompson et al., 2024) and multifocal ERG (mfERG) (Hoffmann et al, 2021).

As the action of portable LT devices is based on retinal function and considering that blue-enriched light can be deleterious to vision, ERG can be used to assess the retinal tolerability of portable LT devices in MDD patients. To our knowledge, no complete or standardized evaluation of the retinal effects of portable LT devices has yet been conducted. Only the effects of conventional LT lamps have been investigated using ffERG. A normalization of ffERG parameters was reported following four weeks of daily 30-minutes LT sessions in patients with Seasonal Affective Disorder (Lavoie et al., 2009). In contrast, Gagné et al. (2007) observed a loss of rod function in ffERG in healthy subjects immediately after a single 60-minutes LT session, an effect likely attributable to the blue-light component of LT (Gagné et al., 2011). However, the ERG changes reported in Gagné’s studies may reflect a transient, physiological adaptation rather than a true deleterious impact on retinal function. Repeated LT sessions would likely be necessary to determine any long-term retinal toxicity.

Therefore, the aim of this study was to assess, using ERG, the impact on retinal function of an 8 weeks exposure to an active or a placebo portable LT device, in combination with usual care, in MDD patients. Given portable LT device mechanism of action is similar to that of conventional LT lamps, we hypothesize that the use of the LT device for 8 weeks would not induce functional alterations – morphological or quantitative – of the retina.

## 2 Patients and methods

### 2.1 Study design

A total of 28 patients, aged over 18 years and diagnosed with MDD according to DSM-IV criteria, were recruited.

Exclusion criteria were as follows:

i) Current progressive psychiatric disorders (excluding MDD and anxiety disorders)
ii) Absence of routine care for MDD
iii) Previous or current LT treatment
iv) Retinal pathology

The LUMIDEP study (NCT03685942), was a randomized, double-blind and placebo-controlled trial designed to evaluate the effects of portable LT on depression severity in addition to usual care. An ancillary study evaluated retinal function of this device. Here, only the results of the ancillary part during baseline and treatment phase are presented but the full study protocol is available online (Cosker et al, 2021).

Eligible patients were randomly assigned to: i) an active treatment group (PT^+^) receiving LT or ii) and a placebo group (PT^−^) receiving a placebo device. Randomization was performed using block randomization with allocation determined by a randomization number.

The trial was conducted at the Psychotherapeutic Center of Nancy (CPN), France, between 2019 and 2023. All procedures were approved by the Ile-de-France X Ethics Committee (protocol number 34–2018), and all participants provided written informed consent.

### 2.2 Light therapy device

**Fig. 1:**
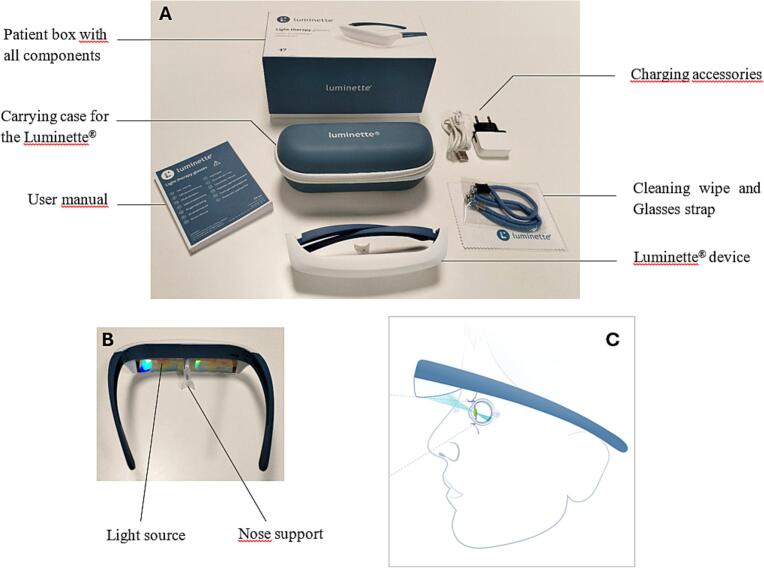
Example of an active Luminette® device and its accessories used in the LUMIDEP study Illustration of the portable light therapy device, the Luminette®, used in the LUMIDEP. study. Both the active and placebo devices were visually identical. (A) Annotated photograph of the Luminette® device and all accessories provided to the patient. (B) Close-up image of the Luminette® showing the holographic visor that emits light. (C) Schematic illustration of the Luminette® being worn, demonstrating how the device directs therapeutic light towards the pupil. This wearable light therapy device allows users to continue their daily activities while receiving their prescribed dose of light therapy.

In the LUMIDEP trial, the portable LT device tested was the Luminette® device (*Lucimed SA, Villers-Le-Bouillet, Belgium;* https://myluminette.com/en-us/products/luminette-2). It is a holographic visor that focuses light towards the pupil. The active device emitted blue-enriched white light with a peak wavelength of 468 nm at an intensity of 1000 lx. The placebo device emitted white light with a peak wavelength of 660 nm at 175 lx, a setting that does not affect the circadian rhythm. Both the active and placebo devices were visually identical and could not be distinguished by either the patients or the researchers. They were worn like a pair of glasses (Fig. 1).

Patients in both groups were instructed to use their assigned device daily alongside their routine care for 30 min, after waking, preferably between 7:00 and 9:00 AM, over a period of 8 weeks.

### 2.3 Procedure

**Fig. 2:**
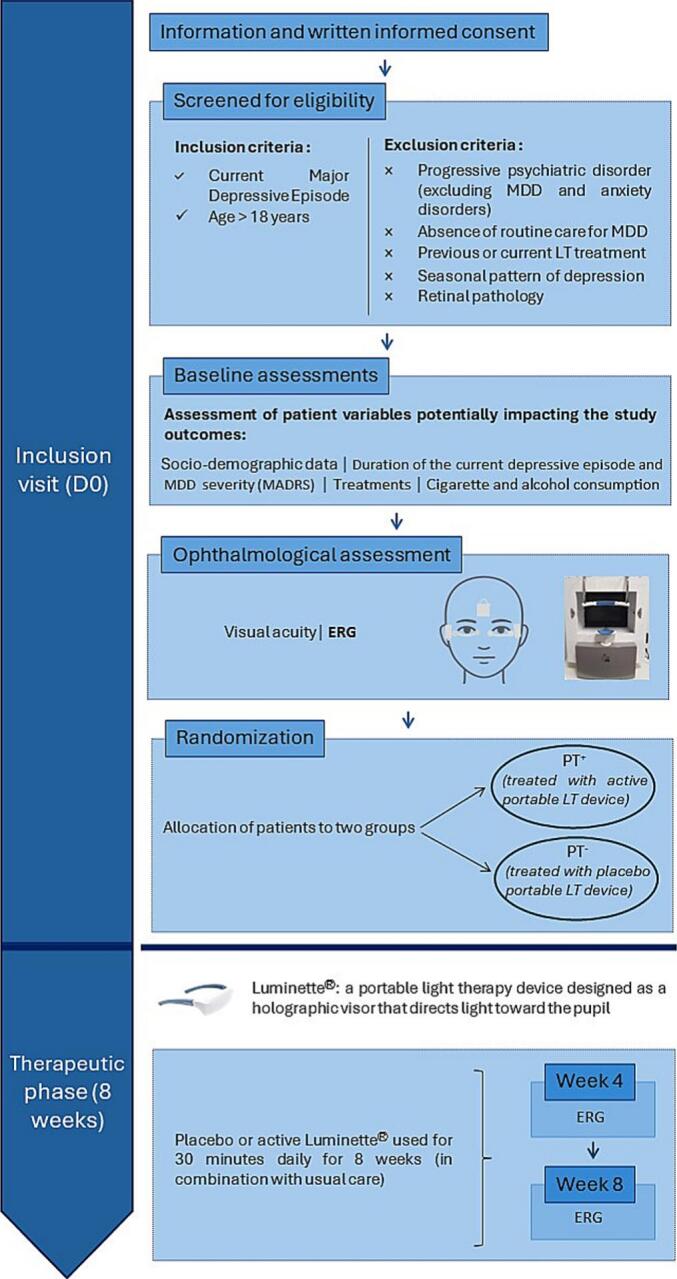
Schematic workflow of the ancillary part of the LUMIDEP study. The ancillary part of LUMIDEP was a randomized, placebo-controlled trial assessing retinal tolerability of a portable light therapy (LT) device in patients with Major Depressive Disorder (MDD) using ElectroRetinoGraphy (ERG). After the inclusion visit at day 0 (baseline), patients started LT treatment the following day (D1). The therapeutic phase lasted 8 weeks, with follow-up assessments conducted at 4 weeks (W4) and 8 weeks (W8) post-inclusion. *Note: D0 = Day 0; ERG = ElectroRetinoGraphy; LT = Light Therapy; MADRS = Montgomery and Asberg Depression Rating Scale; MDD = Major Depressive Disorder*

The overall study workflow is summarized in Fig. 2.

After providing detailed information about the study and obtaining written informed consent, each patient’s eligibility was assessed during the inclusion visit. Psychiatric evaluation was conducted using the MINI 5.0.0 to confirm the diagnosis of MDD and to exclude current progressive psychiatric disorders. Additional inclusion and exclusion criteria were verified through patient interviews.

If the patient was eligible, the severity of depressive symptoms was assessed using the standardized Montgomery and Asberg Depression Rating Scale (MADRS). For the ancillary study, only variables potentially influencing ERG results were analyzed.

Subsequently, patients underwent ffERG, PERG and mfERG assessments. These electrophysiological recordings were conducted according to the standards of the International Society for Clinical Electrophysiology of Vision (ISCEV) (McCulloch et al., 2015, Bach et al., 2013, Hood et al., 2012). Stimuli were presented using the MONPackOne® system associated with the Mon2014D monitor (*Metrovision, Perenchies, France*). Active electrodes were Dawson, Trick & Litzkow (DTL) electrodes placed in the inferior conjunctival sac of the eye. Ground and reference electrodes were skin electrodes positioned at the nasion and between the eyes and ears, respectively. ERG responses were analyzed by extracting the amplitude and implicit time of the characteristic waves specific to each ERG modality (ffERG, PERG, mfERG), in accordance with ISCEV standards.

The day after the inclusion visit, patients began use of the active or placebo device for 8 weeks. Follow-up assessments, including ERG and MADRS evaluations, were conducted at 4 weeks (W4) and 8 weeks (W8) post-inclusion. Only patients who completed at least 45 sessions of LT, each lasting a minimum of 30 min, were included in the statistical analysis.

### 2.4 Statistical analysis

Comparisons between groups were performed with Mann Whitney U tests for quantitative variables and Fisher’s exact tests for qualitative data. The significance level was set at 5 %. Statistical analyses were performed in intention-to-treat using SAS 9.4 software (SAS Inst., Cary, NC, USA).

## 3 Results

### 3.1 Sample description and baseline characteristics

**Fig. 3:**
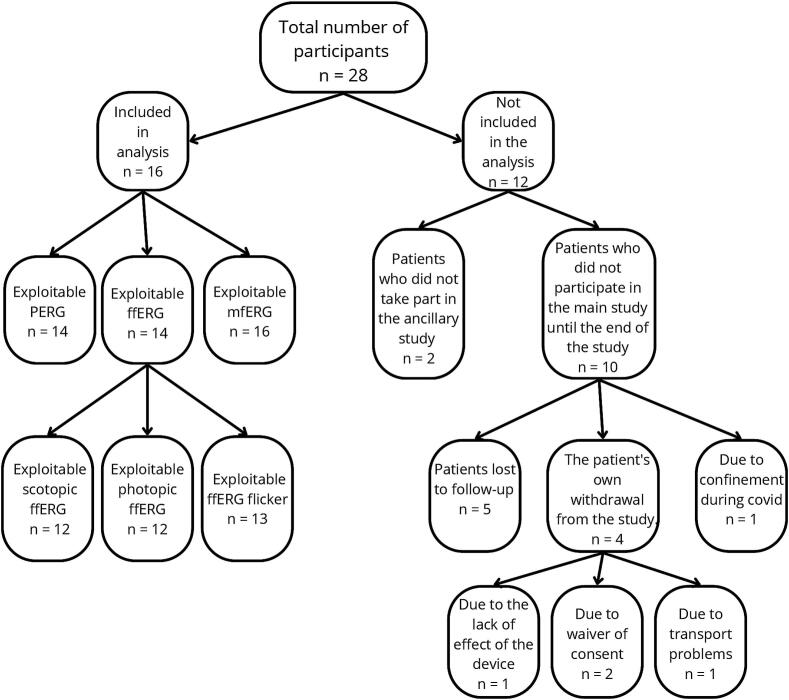
Flow chart illustrating the number of included patients for the ancillary component of the LUMIDEP study. This figure illustrates the flow of participants through the different stages of the LUMIDEP clinical trial, highlighting the loss of patients and the distribution of exploitable data for electroretinography analyses. Firstly, the total number of participants initially recruited into the study was 28. Then, 12 patients were not included in the final analysis. In fact, 2 of these patients did not participate in the ancillary study, while 10 did not participate in the main study until the end. Of these 10 patients, 5 were lost to follow-up, one due to the ineffectiveness of the device. Four patients withdrew from the study of their own volition: two withdrew their consent and one encountered transport problems. Finally, one patient was unable to continue the study due to containment restrictions associated with the COVID-19 pandemic. Thus, 16 patients were included in the final analysis. The exploitable data for the different types of ERG included 14 patients for PERG, 14 patients for ffERG, and 16 patients for mfERG. Regarding ffERG subcategories, 12 patients had usable data for scotopic ffERG, 12 for photopic ffERG, and 13 for flicker ffERG. *Note: ffERG = full-field electroretinography; mfERG = multifocal electroretinography; PERG = Pattern electroretinography.*

Fig. 3 illustrates the participant flow through the different stages of the study. Of the 28 participants enrolled in the LUMIDEP clinical trial, 26 were enrolled in the ancillary study. Among them, 16 completed the study and were therefore included in the subsequent analysis. Due to variations in data quality, the number of participants included in each type of ERG analysis differed slightly and is detailed in Fig. 3.

Baseline socio-demographic, clinical characteristics and substance consumption of these 16 patients are summarized in Table 1. No significant differences were found between groups for any of these baseline characteristics.

**Table 1:** Characteristics potentially influencing ERG results of patients whose ERG were exploitable.

Variables		Patients treated with active Luminette® (N = 6)			Patients treated with placebo (N = 10)			Comparisons PT^+^ vs PT^−^ (P-value)		
Gender (Number of woman/Number of man)		5/1			8/2			1.000^f^		
Age		47.5 (33.0; 53.0)			39.5 (32.0; 46.0)			0.709^m^		
Duration of the current depressive episode (Days)		225.0 (210.0; 365.0)			172.5 (98.0; 330.0)			0.427^m^		
MADRS at inclusion (total score)		29 (19.0; 37.0)			225 (16.0; 27.0)			0.272^m^		
Treatments taken during the study		W0	W4	W8	W0	W4	W8	W0	W4	W8
	SSRI	N = 4	N = 3	N = 3	N = 7	N = 6	N = 6	1.000^f^	1.000^f^	1.000^f^
	SNRI	N = 2	N = 1	N = 1	N = 1	N = 1	N = 2	0.518^f^	1.000^f^	1.000^f^
	TCAD	N = 1	N = 3	N = 3	N = 1	N = 1	N = 1	1.000^f^	0.118^f^	0.118^f^
	Anxiolytics	N = 5	N = 4	N = 5	N = 5	N = 5	N = 5	0.307^f^	0.633^f^	0.307^f^
	Hypnotics	N = 3	N = 3	N = 1	N = 1	N = 1	N = 1	0.118^f^	0.118^f^	1.000^f^
	Antipsychotics	N = 1	N = 0	N = 1	N = 1	N = 1	N = 1	1.000^f^	1.000^f^	1.000^f^
	Lithium carbonates	N = 1	N = 1	N = 1	N = 0	N = 0	N = 0	0.375^f^	0.375^f^	0.375^f^
Fluoxetine dose equivalent of antidepressants (mg/day)		36.7 (20.1; 70.5)	29.6 (25.8; 70.5)	33.9 (20.0; 70.5)	28.4 (22.2; 40.0)	34.5 (22.2; 40.0)	34.8 (20.1; 40.0)	0.523^f^	0.862^f^	1.000^f^
Current cigarette consumption (cigarette packets per year)		1.4 (0.0; 20.0)			0.0 (0.0; 7.0)			0.313		
Current alcohol consumption (Standard units per week)		0.3 (0.0; 7.0)			3.0 (0.0; 10.0)			0.586		

The table summarizes the characteristics of patients potentially influencing ERG results according to “MDD patients treated with an active Luminette® (PT^+^) or “MDD patients treated with a placebo Luminette® (PT^−^) group. The statistical p-value is indicated between the two groups for each data item following i) Mann Whitney U test for quantitative variables, ii) Fisher’s exact test for qualitative data. Data are shown as i) median (1st quartile; 3rd quartile) for quantitative data, ii) Number of patients meeting the condition for qualitative data. * p < 0.05. ^m^ Mann Whitney U test, ^f^ Fisher’s exact test.

*Note: MADRS = Montgomery-Åsberg Depression Rating Scale; PT^+^* = *MDD patients treated with an active Luminette*®*; PT*^−^ = *MDD patients treated with a placebo; SNRI = Serotonin-norepinephrine reuptake inhibitor; SSRI = Selective Serotonin Reuptake Inhibitor; TCAD = Tryciclic antidepressants;*

### 3.2 ElectroRetinoGraphic outcomes

At baseline (D0), no significant differences were observed between the PT^+^ and PT^−^ groups in ffERG, PERG and mfERG parameters (Table 2).

**Table 2:** Baseline results of ERG statistical analyses in MDD patients treated with an active or a placebo Luminette®.

ERG Modality	ERG Parameter	Patients treated with active Luminette®	Patients treated with placebo	Comparisons pt^+^ Vs PT^−^ (P-Value)
Scotopic ffERG (n = 4PT^+^; n = 8PT^−^)	DA0.01 a-wave implicit time (ms)	35.5 (30.9; 37.5)	36.6 (36.2; 37.8)	0.292
	DA0.01 a-wave amplitude (µV)	−9.9 (−13.5; −2.0)	−8.2 (−17.4; −6.3)	0.803
	DA0.01 b-wave implicit time (ms)	73.6 (65.4; 80.9)	77.1 (73.8; 92.0)	0.564
	DA0.01 b-wave amplitude (µV)	83.9 (75.6; 112.8)	157.5 (115.3; 234.8)	0.102
	DA3.0 a-wave implicit time (ms)	21.3 (18.4; 22.2)	20.2 (16.8; 21.5)	0.676
	DA3.0 a-wave amplitude (µV)	−104.2 (−106.5; −92.4)	−129.3 (−165.5; −91.4)	0.370
	DA3.0 b-wave implicit time (ms)	41.3 (40.8; 41.7)	41.0 (39.3; 41.7)	0.676
	DA3.0 b-wave amplitude (µV)	193.3 (153.0; 216.0)	245.5 (187.5; 278.3)	0.229
LA3.0 ffERG (n = 5PT^+^; n = 7PT^−^)	LA3.0 a-wave implicit time (ms)	14.9 (14.9; 15.8)	14.9 (14.5; 15.8)	1.000
	LA3.0 a-wave amplitude (µV)	−17.3 (−18; −14.8)	−18.5 (−21.1; −14.1)	0.351
	LA3.0 b-wave implicit time (ms)	30.0 (30.0; 31.3)	30.9 (30.0; 31.8)	0.808
	LA3.0 b-wave amplitude (µV)	81.8 (81.8; 86.5)	80.4 (51.0; 94.1)	0.529
Flicker ffERG (n = 5PT^+^; n = 8PT^−^)	Flicker 3.0 a-wave implicit time (ms)	15.1 (15.1; 17.3)	16.6 (15.3; 16.8)	1.000
	Flicker 3.0 a-wave amplitude (µV)	−37.5 (−49.0; −34.3)	−48.2 (−62.8; −30.6)	0.830
	Flicker 3.0 b-wave implicit time (ms)	27.8 (27.8; 27.8)	28.5 (27.8; 29.2)	0.696
	Flicker 3.0b-wave amplitude (µV)	77.8 (61.9; 89.3)	82.1 (51.2; 101.9)	0.721
PERG (n = 5PT^+^; n = 9PT^−^)	N35 implicit time (ms)	27.8 (25.6; 30.5)	28.8 (25.6; 32.3)	0.948
	N35 amplitude (µV)	0.3 (−0.3; 0.4)	−0.6 (−1.0; −0.4)	0.119
	P50 implicit time (ms)	52.2 (51.8; 56.1)	51.3 (46.4; 53.9)	0.275
	P50 amplitude (µV)	2.2 (2.1; 2.9)	2.5 (2.0; 3.5)	0.896
	N95 implicit time (ms)	96.0 (91.6; 96.8)	97.3 (92.0; 99.2)	0.648
	N95 amplitude (µV)	−3.5 (−3.6; −2.1)	−4.0 (−5.1; −2.9)	0.205
mfERG (n = 6PT^+^; n = 10PT^−^)	N1 amplitude < 2° (µV)	−553.0 (−895.5; −298.0)	−505.5 (−900.5; −394)	1.000
	N1 implicit time < 2° (ms)	26.1 (25.9; 28.3)	27.8 (24.7; 30.7)	0.790
	N1 amplitude 2-5° (µV)	−201.8 (−251.0; −173.3)	−271.5 (−298.0; −134.0)	0.873
	N1 implicit time 2-5° (ms)	27.3 (26.5; 28.4)	25.9 (24.9; 27.3)	0.231
	N1 amplitude 5-10° (µV)	−152.1 (−169.0; −140.4)	−212.3 (−266.0; −134.2)	0.790
	N1 implicit time 5-10° (ms)	25.9 (24.7; 26.8)	25.6 (24.4; 26.8)	0.873
	N1 amplitude 10-15° (µV)	−168.3 (−179.0; −131.0)	−242.5 (−275.0; −182.0)	0.137
	N1 implicit time 10-15° (ms)	25.1 (24.0; 25.7)	25.0 (23.8; 25.9)	0.958
	N1 amplitude > 15° (µV)	−171.3 (−201.0; −167.5)	−194.8 (−225.3; −130.5)	1.000
	N1 implicit time > 15° (ms)	24.8 (24.7; 25.3)	25.0 (24.0; 25.8)	0.595
	P1 amplitude < 2° (µV)	766.8 (677.5; 1179.0)	862.8 (599.5; 1116.5)	0.958
	P1 implicit time < 2° (ms)	50.8 (50.4; 51.6)	49.8 (47.3; 51.1)	0.295
	P1 amplitude 2-5° (µV)	485.3 (404.5; 591.5)	456.5 (324.0; 572.5)	0.429
	P1 implicit time 2-5° (ms)	46.6 (45.8; 47.4)	45.8 (45.2; 47.7)	0.371
	P1 amplitude 5-10° (µV)	355.3 (313.2; 430.0)	423.3 (293.0; 550.0)	0.790
	P1 implicit time 5-10° (ms)	43.8 (43.2; 44.5)	43.9 (42.1; 45.5)	0.790
	P1 amplitude 10-15° (µV)	363.0 (341.0; 469.0)	468.8 (311.5; 545.5)	0.790
	P1 implicit time 10-15° (ms)	43.3 (42.8; 43.8)	43.7 (43.3; 44.8)	0.251
	P1 amplitude > 15° (µV)	428.0 (390.5; 501.0)	416.3 (320.0; 553.0)	0.710
	P1 implicit time > 15° (ms)	43.0 (42.8; 43.8)	43.8 (43.5; 45.3)	0.319

The table summarizes the ERG results at inclusion visit for patients in the “MDD patients treated with an active Luminette® (PT^+^) or “MDD patients treated with a placebo Luminette® (PT^−^) group. The statistical p-value is indicated between the two groups for each data item following a Mann Whitney U test. Data correspond to the average of the two eyes for each variable and are shown as median (1st quartile; 3rd quartile).

Note: ffERG = Full-Field ElectroRetinoGraphy; mfERG = Multifocal ElectroRetinoGraphy; PERG= Pattern ElectroRetinoGraphy; PT^+^ = MDD patients treated with an active Luminette®; PT^−^ = MDD patients treated with a placebo.

During the treatment phase, no significant differences were observed between groups at mid-treatment (W4) and at the end of the treatment (W8) for: i) a- and b- wave amplitudes or implicit times in ffERG; ii) N35, P50 and N95 wave parameters in PERG − although a statistical trend was noted for N95 implicit time at W8 (p = 0.052, Mann Whitney *U* test); and iii) N1 and P1 amplitudes or implicit times in any concentric retinal regions (<2°, 2-5°, 5-10° and 10-15° around the fovea) in mfERG (Table 3).

**Table 3:** Results of ERG statistical analyses in MDD patients treated with an active or a placebo Luminette® during treatment phase.

ERG Modality	ERG Parameter	Patients treated with active Luminette®		Patients treated with placebo		Comparisons PT^+^ Vs PT^−^ (P-Value)	
		W4	W8	W4	W8	W4	W8
Scotopic ffERG (n = 4PT^+^; n = 8PT^−^)	DA0.01 a-wave implicit time (ms)	35.1 (33.3; 36.6)	34.2 (31.8; 35.8)	35.5 (28.4; 38.2)	35.9 (34.4; 36.9)	1.000	0.194
	DA0.01 a-wave amplitude (µV)	−3.2 (−10.5; 4.2)	−15.4 (−19.3; −8.4)	−14.0 (−16.6; −3.5)	−12.1 (−17.6; −9.6)	0.413	0.679
	DA0.01 b-wave implicit time (ms)	74.0 (67.6; 79.1)	77.1 (71.8; 81.1)	73.8 (67.2; 78.5)	71.8 (63.8; 76.9)	1.000	0.370
	DA0.01 b-wave amplitude (µV)	134.7 (97.3; 192.0)	117.6 (93.4; 148.5)	153.5 (103.9; 225.0)	134.5 (125.3; 166.3)	0.804	0.370
	DA3.0 a-wave implicit time (ms)	21.5 (18.9; 22.0)	21.3 (19.1; 21.8)	18.5 (16.5; 22.0)	18.5 (17.6; 20.4)	0.619	0.459
	DA3.0 a-wave amplitude (µV)	−119.5 (−139.3; −82.0)	−96.3 (−117.0; −71.9)	−120.2 (−174.0; −74.6)	−120.5 (−153.5; −99.9)	0.804	0.293
	DA3.0 b-wave implicit time (ms)	38.8 (36.2; 40.6)	39.3 (38.6; 44.6)	40.4 (39.3; 41.3)	40.8 (39.5; 42.4)	0.459	0.510
	DA3.0 b-wave amplitude (µV)	224.3 (149.4; 275.8)	190.0 (147.5; 207.8)	211.0 (188.3; 304.0)	244.0 (205.8; 289.5)	0.804	0.135
LA3.0 ffERG (n = 5PT^+^; n = 7PT^−^)	LA3.0 a-wave implicit time (ms)	14.9 (14.9; 14.9)	15.3 (14.9; 15.3)	14.9 (14.5; 15.3)	14.9 (14.5; 16.3)	1.000	0.873
	LA3.0 a-wave amplitude (µV)	−17.4 (–22.1; −13.8)	−15.1 (−15.7; −13.8)	−15.7 (−25.4; −13.4)	−19.8 (−21.3; −16.2)	1.000	0.077
	LA3.0 b-wave implicit time (ms)	30.9 (30.0; 30.9)	30.9 (30.0; 31.3)	30.0 (29.1; 32.6)	30.4 (29.5; 32.6)	0.810	0.936
	LA3.0 b-wave amplitude (µV)	86.1 (79.6; 107.2)	84.1 (81.7; 87.3)	84.6 (75.6; 118.0)	87.1 (62.3; 101.5)	1.000	0.751
Flicker ffERG (n = 5PT^+^; n = 8PT^−^)	Flicker 3.0 a-wave implicit time (ms)	15.0 (14.6; 18.6)	16.3 (14.2; 16.4)	16.3 (14.2; 16.6)	15.0 (14.6; 17.0)	0.773	0.774
	Flicker 3.0 a-wave amplitude (µV)	−42.5 (−50.0; −41.7)	−38.1 (−40.0; −38.0)	−41.8 (−54.9; −37.2)	−44.8 (−55.3; −34.8)	0.830	0.523
	Flicker 3.0 b-wave implicit time (ms)	28.3 (27.8; 28.8)	28.3 (27.4; 30.1)	28.3 (27.4; 29.6)	28.8 (27.6; 29.2)	0.942	1.000
	Flicker 3.0b-wave amplitude (µV)	85.6 (44.5; 94.0)	64.0 (61.3; 70.1)	78.2 (59.1; 91.3)	76.5 (65.6; 89.8)	0.721	0.190
PERG (n = 5PT^+^; n = 9PT^−^)	N35 implicit time (ms)	20.8 (19.4; 23.4)	27.3 (25.6; 27.8)	27.4 (22.9; 31.3)	24.3 (23.4; 28.3)	0.067	0.517
	N35 amplitude (µV)	−0.5 (−0.5; −0.3)	0.0 (−0.6; 0.0)	−0.3 (−1.1; 0.1)	−0.5 (−1.2; 0.0)	0.948	0.517
	P50 implicit time (ms)	52.2 (50.8; 56.1)	52.6 (51.8; 53.9)	53.0 (49.9; 54.4)	53.9 (47.3; 54.8)	0.896	0.648
	P50 amplitude (µV)	3.3 (2.5; 3.3)	2.4 (2.3; 2.5)	2.8 (1.8; 3.4)	2.4 (2.3; 3.5)	0.896	0.696
	N95 implicit time (ms)	101.1 (99.1; 103.0)	91.1 (91.1; 99.1)	101.4 (90.7; 106)	106.5 (102.2; 113)	1.000	0.052
	N95 amplitude (µV)	−3.2 (−4.3; −3.0)	−2.9 (−4.0; −2.6)	−3.4 (−4.0; −2.9)	−3.8 (−4.7; −2.8)	0.794	0.844
mfERG (n = 6PT^+^; n = 10PT^−^)	N1 amplitude < 2° (µV)	−597.8 (−686.0; −535.0)	−428.5 (−522.0; −196)	−459.0 (−689.5; −310.5)	−615 (−889.0; −536.0)	0.319	0.077
	N1 implicit time < 2° (ms)	28.6 (25.8; 29.4)	28.8 (25.8; 30.9)	28.5 (26.4; 33.3)	28.9 (27.0; 31.1)	0.710	0.958
	N1 amplitude 2-5° (µV)	−231.5 (−359.0; −211.0)	−267.3 (−293.5; −248.0)	−271.3 (−338.0; −250.5)	−288.0 (−346.5; −186.0)	0.790	0.958
	N1 implicit time 2-5° (ms)	27.3 (24.8; 28.1)	26.4 (25.4; 26.9)	26.7 (25.3; 28.9)	27.3 (26.2; 28.6)	0.831	0.560
	N1 amplitude 5-10° (µV)	−245.0 (−308.0; −192.0)	−204.5 (−236.5; −120.5)	−206.0 (−276.5; −178.0)	−207.0 (−380.0; −120.0)	0.710	1.000
	N1 implicit time 5-10° (ms)	25.7 (24.5; 26.2)	24.3 (23.9; 25.8)	26.1 (24.3; 27.2)	26.6 (25.4; 28.6)	0.831	0.231
	N1 amplitude 10-15° (µV)	−181.0 (−320.5; −147.0)	−208.0 (−256.8; −142.5)	−173.3 (−212.0; −139.0)	−172.5 (−211.0; −129.5)	0.873	0.492
	N1 implicit time 10-15° (ms)	26.2 (24.5; 28.4)	25.5 (25.4; 28.3)	25.9 (24.6; 27.2)	25.6 (23.9; 26.8)	0.958	0.429
	N1 amplitude > 15° (µV)	−175.7 (−236.0; −145.6)	−169.8 (−204.5; −121.0)	−210.8 (−257.0; −159.5)	−155.1 (−282.5; −111.4)	0.710	0.790
	N1 implicit time > 15° (ms)	25.0 (24.2; 26.3)	25.1 (24.8; 25.5)	25.5 (24.4; 27.3)	25.9 (25.3; 27.1)	0.749	0.319
	P1 amplitude < 2° (µV)	864.5 (800.5; 1021.0)	879.3 (723.0; 1130.0)	1082.5 (494.5; 1342.5)	1005.5 (736.5; 1447.5)	0.958	0.710
	P1 implicit time < 2° (ms)	50.0 (44.8; 51.7)	51.1 (49.4; 53.8)	52.8 (51.3; 58.9)	52.6 (51.5; 53.1)	0.094	0.524
	P1 amplitude 2-5° (µV)	545.8 (417.5; 704.0)	525.0 (356.0; 623.0)	511.0; 381.5; 617.0)	554.0 (314.5; 664.0)	0.633	1.000
	P1 implicit time 2-5° (ms)	46.2 (45.7; 50.1)	45.9 (45.1; 47.5)	46.4 (45.6; 47.7)	46.8 (46.1; 48.0)	1.000	0.319
	P1 amplitude 5-10° (µV)	434.3 (383.0; 596.0)	358.3 (236.5; 494.5)	407.8 (308.0; 541.5)	358.5 (326.0; 610.5)	0.399	0.873
	P1 implicit time 5-10° (ms)	43.5 (42.9; 45.5)	44.4 (42.8; 45.5)	44.8 (43.4; 45.8)	45.0 (44.0; 45.8)	0.399	0.429
	P1 amplitude 10-15° (µV)	332.5 (258.0; 411.5)	361.3 (218.0; 436.5)	364.0 (341.0; 415.5)	391.8 (257.0; 522.5)	0.560	0.790
	P1 implicit time 10-15° (ms)	43.1 (41.9; 43.4)	42.7 (41.8; 43.1)	44.8 (43.7; 46.5)	44.0 (43.2; 45.8)	0.077	0.077
	P1 amplitude > 15° (µV)	393.0 (357.0; 450.5)	356.3 (277.0; 460.0)	425.3 (319.0; 528.0)	376.0 (218.5; 550.0)	0.873	0.873
	P1 implicit time > 15° (ms)	43.9 (42.9; 45.8)	43.7 (42.9; 44.6)	44.8 (42.3; 45.8)	44.8 (43.0; 45.3)	0.915	0.399

The table summarizes the ERG results during treatment phase, i.e at 4 (W4) and 8 weeks (W8) after inclusion visit for patients in the “MDD patients treated with an active Luminette® (PT^+^) or “MDD patients treated with a placebo Luminette® (PT^−^) group. The statistical p-value is indicated between the two groups for each data item following a Mann Whitney U test. Data correspond to the average of the two eyes for each variable and are shown as median (1st quartile; 3rd quartile).

Note: ffERG = Full-Field ElectroRetinoGraphy; mfERG = Multifocal ElectroRetinoGraphy; PERG= Pattern ElectroRetinoGraphy; PT^+^ = MDD patients treated with an active Luminette®; PT^−^ = MDD patients treated with a placebo.

## 4 Discussion

### 4.1 Results interpretation

This study aimed to assess the retinal function after 8 weeks use of a portable LT device, in MDD patients using ERG.

Following the results analysis, no significant differences were found in the ERG waveforms between the patients using active Luminette® (PT^+^) and patients using the placebo device (PT^−^) after 4 and 8 weeks.

Overall, these results show that 8 weeks of daily exposure to Luminette®, combined with usual care, does not lead to retinal function alterations in our cohort of MDD patients. This outcome is consistent with our initial hypothesis, based on literature, that portable LT devices are unlikely to induce retinal alterations when used appropriately (Brouwer et al., 2017, Lavoie et al., 2009). However, several limitations constrain the interpretation of our findings.

### 4.2 Retinal safety of potable LT devices: Mechanistic insights and new approach methodologies

While ERG is a standard method for evaluating retinal function, the electrooculogram (EOG) also offers valuable insights (Robson et al., 2018). This electrophysiological test measures the standing potential between the cornea and the retina during prolonged light and dark adaptation. It provides complementary information, particularly regarding retinal pigment epithelium function. EOG has previously been used to demonstrate retinal safety following LT exposure (Ozaki et al., 1993).

Beyond retinal function, potential structural impact should also be considered, as structural retinal damage has been reported following blue-light exposure (Li et al., 2021, Nagai et al., 2015). To evaluate such effects, structural ophthalmologic imaging techniques may be valuable. Fundus photography provides a general visual assessment of the retina, while Optical Coherence Tomography (OCT) offers imaging of retinal layers. Both techniques have demonstrated no structural retinal damage from conventional LT lamps (Sloane et al., 2015, Rosenthal, 1984, Fukuda et al., 1998, Gallin et al., 1995). Therefore, these imaging approaches could likewise be applied to portable LT devices.

However, these methods can be difficult to implement and may cause discomfort, particularly in psychiatric patients. This is the case with ERG assessments involving DTL electrodes, which can be uncomfortable to wear due to their placement in the conjunctival sac of the eye. Pupil dilatations also causes discomfort, impairing temporarily vision and preventing activities such as driving. Therefore, although investigating the effects of LT on retinal function in human subjects remains essential, in vitro and in silico approaches could serve as a preliminary step to better identify specific retinal alterations associated with LT exposure. They may help refine ERG protocols by targeting relevant retinal parameters and thereby reduce the number and duration of invasive procedures required in clinical trials. In addition, advanced in vitro and in silico platforms also offer valuable tools to explore potential photochemical damage caused by blue-enriched LT (Ouyang et al., 2020, Tosini et al., 2016).

For instance, live retinal explants systems enable real-time monitoring of cellular damage − such as photoreceptor injury − and reactive oxygen species (ROS) generation following blue-enriched LT exposure (Roehlecke et al., 2011). Retinal pigment epithelium (RPE) cultures and human retinal organoids derived from human-induced pluripotent stem cells (hiPSCs) allow for a more human-relevant analysis of cellular stress pathways, including blue light-induced mitochondrial dysfunction, apoptosis and inflammatory pathways (Chakravarthy et al., 2024, Xu et al., 2023).

Finally, a computational in silico model has recently been developed to simulate retinal physiology in response to light exposure (Abuelnasr and Stinchcombe, 2023). Although this model has not yet been applied to evaluate blue-light-induced retinal damage, it holds potential for future use in mechanistic risk assessment of light-based therapies. To enable such applications, further developments are required – notably the incorporation of phototoxicity mechanisms and oxidative stress pathways, for example through the integration of a RPE module capable of representing the biochemical responses to light-induced stress.

Integrating these new approach methodologies will be essential to develop a more comprehensive and ethical framework for evaluating the long-term retinal safety of portable LT devices. These platforms not only enable the investigation of different types of retinal alterations but also allow for the integration of multiple variables that may influence retinal outcomes. This is particularly important for distinguishing the specific contribution of the LT itself from other potentially interacting factors. One relevant example involves psychotropic medications, which are part of standard care for MDD and may be used alongside LT (Menegaz de Almeida et al, 2025). Psychotropic drugs have been linked to ocular toxicity (Richa and Yazbek, 2010), including antipsychotics (Balogun and Coker, 2024), antidepressants − especially Selective Serotonin Reuptake Inhibitors (SSRIs) (Moulard et al., 2022, Guclu et al., 2018) − and mood-stabilizing antiepileptics (Hu et al., 2022). Particular attention should be paid to psychotropic drugs with photosensitizing properties, notably within the blue-light spectrum, such as lamotrigine (Bilski et al., 2009). These properties could theoretically increase retinal susceptibility to light-induced damage when combined with LT. Although our current findings did not reveal evidence of retinal toxicity, it remains possible that future studies could identify subtle or cumulative retinal alterations attributable, at least in part, to concomitant pharmacological treatment. New approach methodologies provide a promising avenue to investigate the independent and synergistic effects of psychotropic medications in combination with LT. Such approaches could refine mechanistic insights and enhance the robustness of retinal safety evaluations in clinical populations.

### 4.3 Study limitations

One of the limitations of this study lies in the small sample size analyzed for each ERG modality. Depending on the ERG condition, only 4 to 10 participants per group were analyzed, increasing variability both within and between groups. This small sample size raises the risk of type II errors (Button et al., 2013), meaning that potentially meaningful differences in retinal function between the active and placebo LT groups may have gone undetected. Moreover, low-powered studies are more susceptible to sampling noise and unstable effect size estimated, which limits the generalizability and reproducibility of the findings (Button et al., 2013). This makes it difficult to draw firm conclusions, particularly when statistical trends are observed, such as the one observed for N95 implicit time (p = 0.052). Thus, the small sample size in this study compromises the reliability of our conclusions. Future studies should aim for adequate sample sizes to more confidently assess the retinal safety of portable LT devices.

Additionally, another important limitation of the present study is its exclusive focus on functional retinal assessment. Integrating complementary methods – such as mechanistic assays with in vitro and in silico methods – would also provide a more comprehensive safety profile of portable LT devices.

Another limitation concerns the method used to monitor LT adherence, which relied on self-reported diaries completed by participants. This subjective approach is prone to inaccuracies, whether due to unintentional misreporting or incorrect use of the LT device – factors that could not be verified through this approach. To enhance the reliability and precision of LT adherence data, future studies should consider implementing objective monitoring systems, such as integrated usage-tracking modules in the Luminette® device. Despite these limitations, it is important to note that the LT devices used in this study were specifically engineered for research and operated exclusively in a standardized mode (intensity level 2). This ensured consistent light exposure across all participants, thereby minimizing variability in LT administration.

### 4.4 Conclusion

To conclude, eight weeks of treatment with the Luminette® device did not lead in any detectable alterations in retinal function. While these preliminary findings support the retinal safety of Luminette® in our cohort of MDD patients, the limited sample size and study constraints warrant cautious interpretation. These results represent a first step toward establishing the ocular safety profile of portable LT devices in clinical populations. Further studies involving larger cohorts and complementary assessment methods are essential to validate these results and investigate potential long-term effects of LT. Particular emphasis should be placed on integrating new approach methodologies to provide mechanistic insights into potential retinal phototoxicity associated with portable LT devices.

## Funding/support

This work was mainly funded by the Psychotherapic Center of Nancy [*Grant* ID  RIPH 2017-02] with financial support from Lucimed.

## CRediT authorship contribution statement

**Marie de Deus:** Data curation, Formal analysis, Writing – original draft. **Charlotte Petit:** Data curation, Formal analysis, Writing – original draft. **Eve Cosker:** Data curation, Investigation, Project administration, Writing – review & editing. **Amandine Luc:** Formal analysis, Writing – review & editing. **Cédric Baumann:** Methodology, Formal analysis, Writing – review & editing. **Thomas Schwitzer:** Funding acquisition, Investigation, Supervision, Writing – review & editing.

## Declaration of competing interest

The authors declare the following financial interests/personal relationships which may be considered as potential competing interests: The portable bright-light therapy devices were provided by the company Lucimed SA *(Villers-Le-Bouillet, Belgium*) and the study was partially financed by this company. However, Lucimed was not involved in the collection and analysis of data, nor in the manuscript preparation.

## Data Availability

Data will be made available on request.

## Glossary

Ancillary research: a supplementary research project to a study, which is independent but uses samples and/or data from subjects in the main study.
Circadian rhythm: a cyclical biological process lasting around 24 hours.
Declarative approach: an approach based on a person's own words to gather data.
Double-blind: clinical trials in which neither the patient nor the investigator knows which treatment alternative the patient is taking.
DTL electrodes: These metal-coated fiber electrodes are widely used as active electrodes in electroretinography. They are placed in the lower conjunctival cul-de-sac, i.e. under the eyelid.
Electroretinography: an electrophysiologic technique which allows to assess the function of the different retinal cells thanks to electrodes.
International Society for Clinical Electrophysiology of Vision: an organization which publishes minimum standards for routine visual examinations, enabling comparisons between laboratories and relevant literature searches.
Light therapy: a medical treatment widely used in psychiatry to treat circadian rhythm disorders and/or depression. It involves exposing the eyes to light similar to sunlight.
Montgomery-Åsberg Depression Rating Scale: a standardized clinical scale, in the form of a questionnaire, which assesses the intensity of depressive symptoms in a patient.
Monocentric approach: clinical trials in which a single research center recruits and includes patients in the study.
Placebo: a treatment that has no specific efficacy of its own, but has the same appearance as the active treatment actually tested.
Pupillary dilatation: an increase in pupil diameter, which occurs naturally to adapt the eye to darkness, but can also be induced by specific products.
Randomisation: an allocation method based on a random process.
